# Ileal Obstruction Secondary to Internal Hernia Under the External Iliac Artery: A Case Report and Literature Review

**DOI:** 10.7759/cureus.28107

**Published:** 2022-08-17

**Authors:** Prashanth B Chowdary, Christopher Wright

**Affiliations:** 1 Colorectal Surgery, Maidstone and Tunbridge Wells NHS Trust, Tunbridge Wells, GBR

**Keywords:** “conservative or therapeutic or gastrograffin or medical and surgical management or surgical or open or laparoscopic and adhesive small bowel obstruction or intestinal obstruction, strangulated internal hernia, explorative laparotomy, pelvic lymphadenectomy, mechanical intestinal obstruction

## Abstract

Bowel obstruction is one of the most common causes of surgical admission. Most of these patients are managed with non-operative management, often resolving 24 to 48 hours after admission. If conservative management fails in patients with adhesional bowel obstruction, surgery is usually the only other option. Surgery often involves the division of adhesions and resection of the non-viable intestine. Occasionally, unexpected findings require quick but safe interventions, including discussions with other specialities.

This report presents a patient who had previously undergone robotic radical cystectomy, pelvic lymphadenectomy and ileal conduit formation. He was admitted with bowel obstruction and failed conservative management. During laparotomy, a loop of ileum had herniated under the right external iliac artery and was ischaemic necessitating resection-anastomosis. We discuss the management of this patient and the available literature regarding this rare presentation.

## Introduction

Bowel obstruction presenting as an emergency is commonplace in a surgical department. Most cases of bowel obstruction will be managed using conservative measures, failing which they undergo surgery. Here, we discuss a patient who presented in obstruction and underwent laparotomy to reveal internal herniation of the small bowel under the previously dissected right external iliac artery.

## Case presentation

A 74-year-old gentleman presented to the Accident and Emergency Department with a 12-hour history of spasmodic abdominal pain and multiple episodes of vomiting. He had passed stool that morning after a suppository; however, the pain persisted. There was no documented fever. He had a history of robotic radical cystectomy and pelvic lymphadenectomy with ileal conduit formation four years ago. Two years ago, he was admitted with urosepsis requiring him to have temporary bilateral nephrectomies. He was also known to have chronic constipation. He was otherwise fit for his age and independent with activities of daily living.

On examination, he had a pulse rate of 82 per minute, respiratory rate of 16 cycles per minute, oxygen saturation was 96% on room air, the oral temperature was 36.8 degrees Celsius, and blood pressure was 180/72 mm Hg, and this was attributed to the pain. The overall National Early Warning Score 2 was 0. His abdomen was distended. No tenderness was noted on palpation of the abdomen, and bowel sounds were present. The ileal conduit was functional. Examination of the inguinal region revealed no evidence of a hernia. There were no abnormalities noted in the review of other organ systems. His venous blood gas was normal, apart from lactate of 2.1 mmol/L. The urine dip test showed trace leucocytes and protein. His complete blood count showed a haemoglobin of 145 g/dl, white cell count of 11.97 cells/mL, and platelet count of 161 x 103 cells/mL. The creatinine level was 119 µmol/L indicating stage I acute kidney injury (AKI). The C-reactive protein level and serum albumins were 15 mg/L and 42 g/L, respectively.

**Figure 1:**
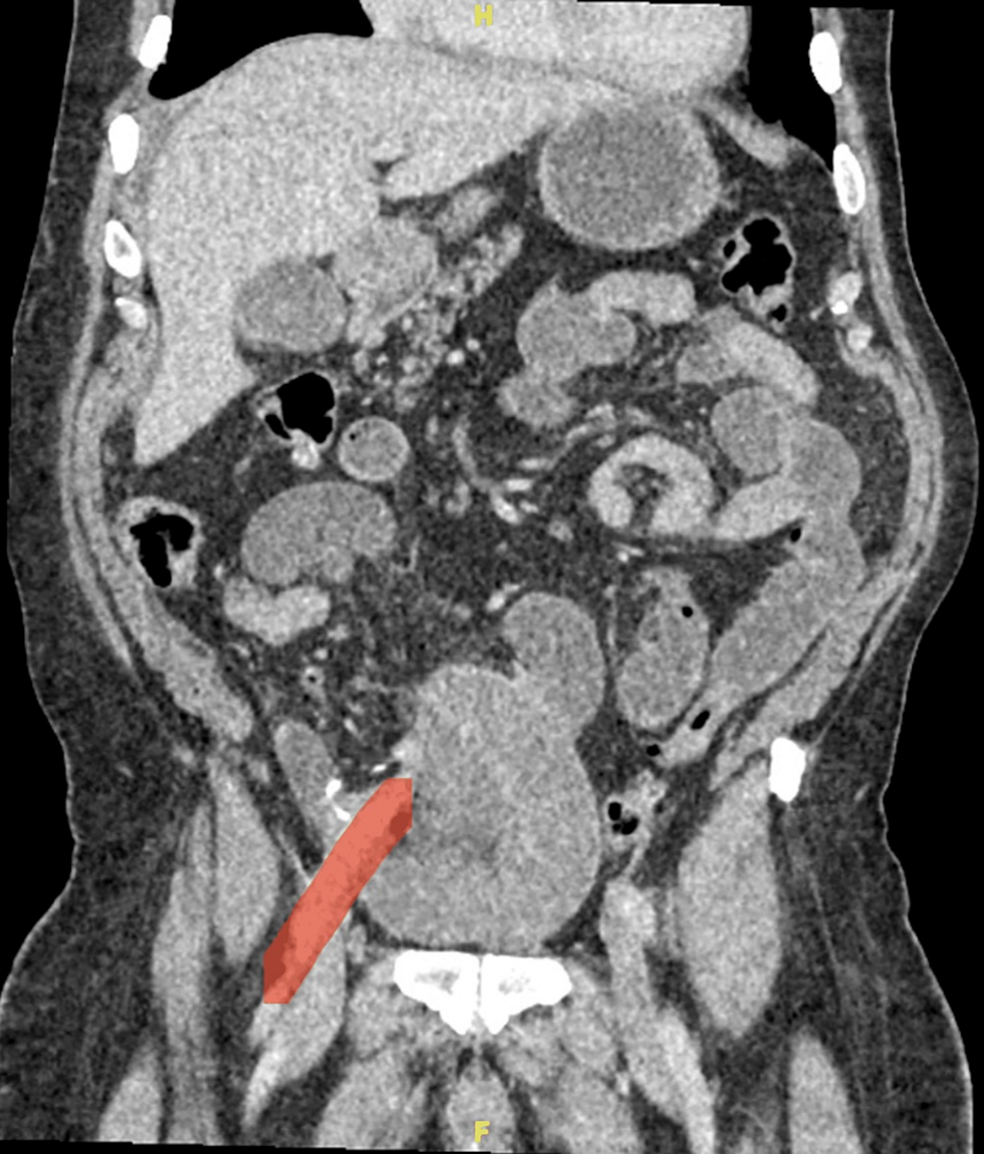
Dilated bowel loops adjacent to the right external iliac artery (marked in red).

**Figure 2:**
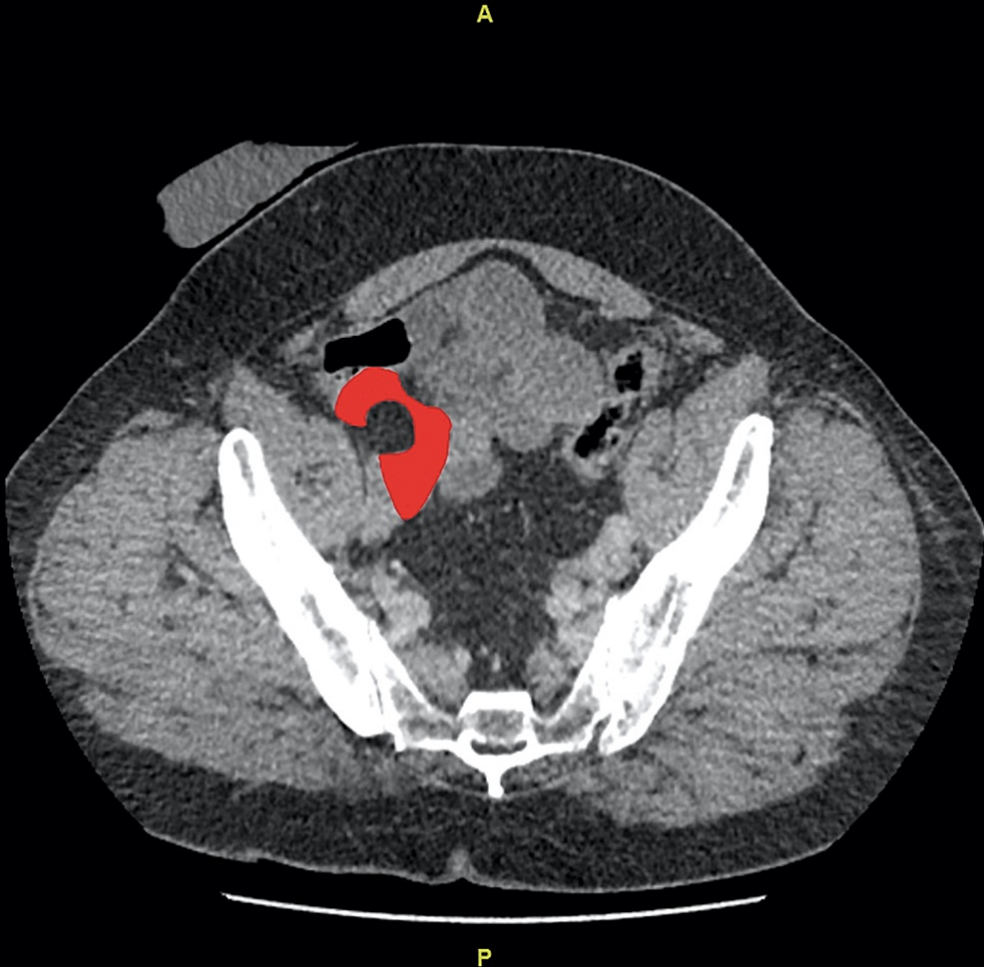
Redundant dissected right external iliac artery (marked in red) with the hernial defect containing fat (noted retrospectively).

With a provisional diagnosis of bowel obstruction secondary to adhesions and acute kidney injury, a nasogastric tube (NG) was inserted, and intravenous fluids and analgesia were administered. A contrast-enhanced computed tomography (CECT) scan of the abdomen and pelvis was performed to confirm the diagnosis, identify the level of obstruction and look for signs necessitating immediate surgery. The CECT scan showed small bowel obstruction (SBO) features with no clear transition point (Figures 1, 2). Following the CECT scan, an initial conservative approach was suggested for 48 hours, failing which a laparotomy would be required.

The following day, the obstruction had not resolved, and 100 ml of gastrograffin was administered through the NG tube. Unfortunately, the patient vomited this, and a decision to leave the NG tube on free drainage and aspirate it every four hours was taken. The obstruction failed to resolve 48 hours after admission, and we decided to perform a laparotomy. This operation's calculated p-possum morbidity and mortality were 91.1% and 6.2%, respectively. Arterial blood gas analysis performed at the time of decision for surgery was normal, apart from a pO2 of 8.5 mmol/L and lactate level of 1.8 mmol/L.

**Figure 3:**
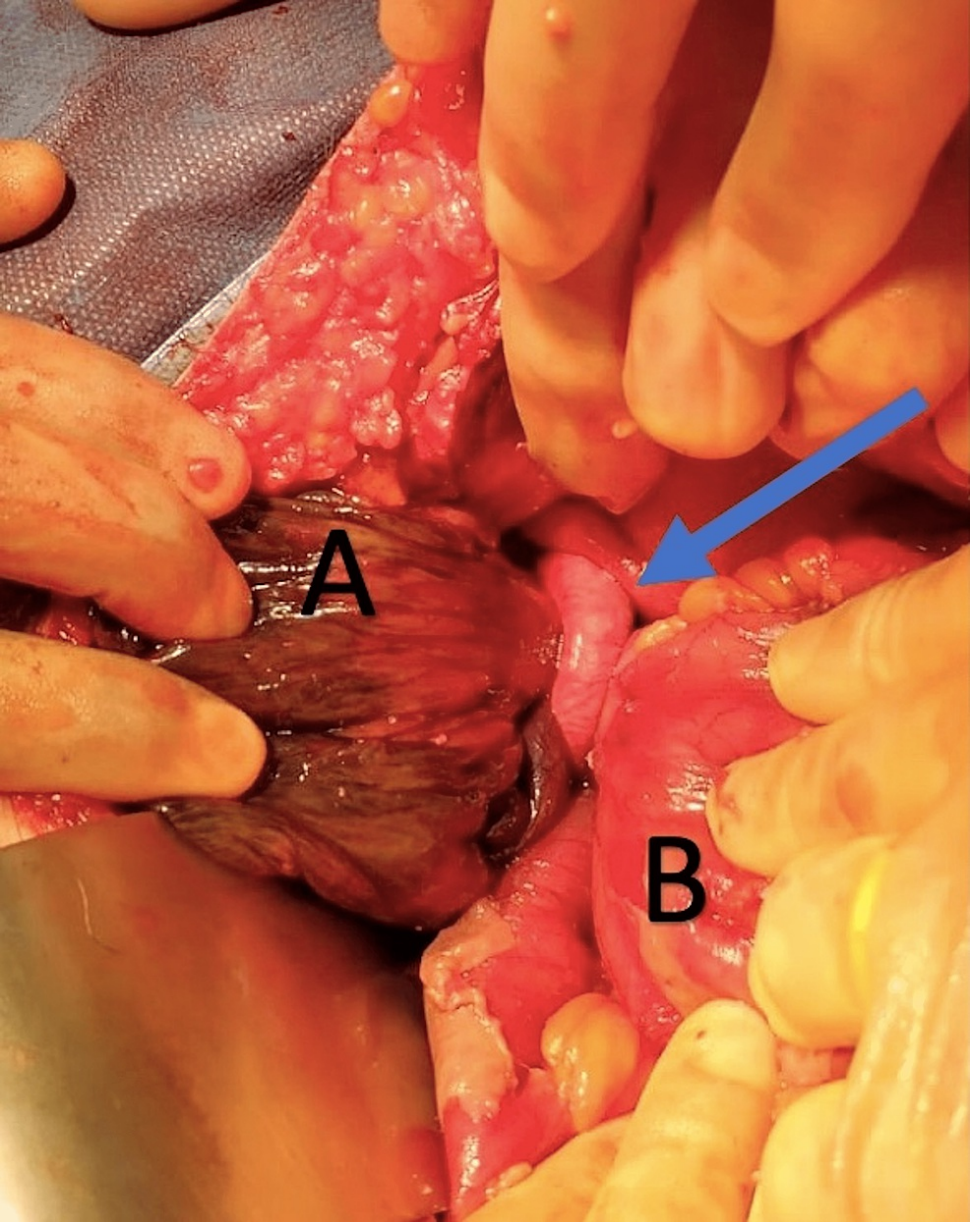
A - Herniated necrotic closed loop of ileum with its mesentery, B - healthy proximal ileum. Previously dissected right external iliac artery (blue arrow).

**Figure 4:**
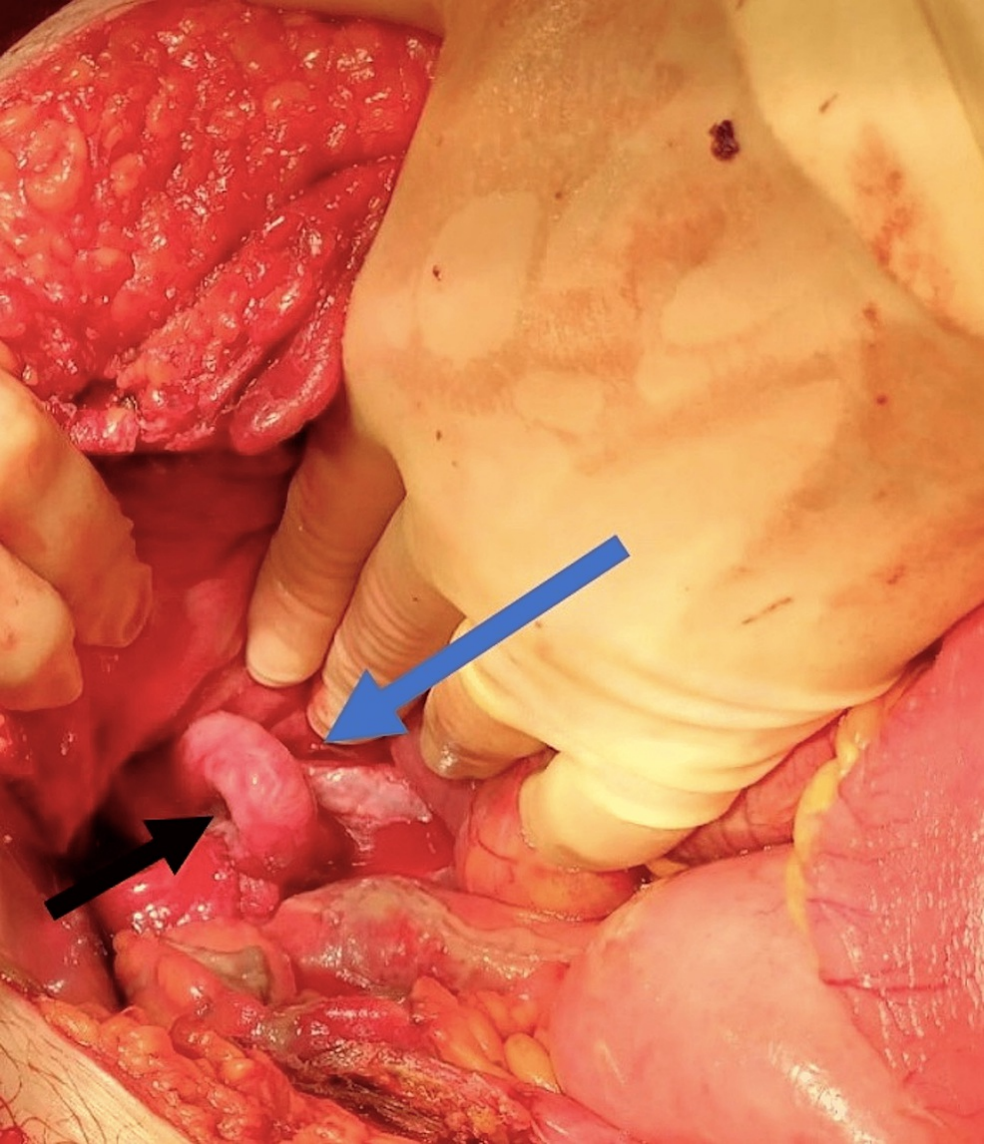
Right external artery after resection of the necrotic closed loop of the ileum (blue arrow) and hernial orifice (black arrow).

He underwent a midline laparotomy, which showed a 7-cm loop of ileum 20 cm proximal to the ileocaecal junction to have herniated under the right external iliac artery and was gangrenous, having formed a closed loop (Figure 3). Due to congestion and inflammatory changes, the bowel loop was stuck, and it was impossible to safely release the bowel despite performing an enterotomy to empty the bowel content. The ileal conduit appeared not to be involved, and a palpable thrill was noted in the external iliac artery, proximal and distal to the herniated bowel. After discussing with a Vascular surgeon and a Urologist, the dead bowel was resected (Figure 4), freeing two ends of the closed loop. A side-to-side ileo-ileal anastomosis was performed using staplers, and the stapled ends were oversewn. A thorough washout was performed, Robinson's drain was left in the pelvis, and a 16 Fr Foley catheter was placed into the ileal conduit as a precaution in case the patient required contrast imaging for an inadvertent injury.

The patient was extubated post-operatively and moved to the intensive care unit for further management. His bowel function returned on postoperative day 4. His AKI was resolved within one week of the surgery. The patient developed a superficial surgical site infection over the lower aspect of the wound, and a vacuum-assisted closure device was placed. He was discharged two weeks after the operation with a plan of clinic follow-up in four weeks.

## Discussion

The most common cause of bowel obstruction is post-operative or congenital adhesions [1]. Internal hernia causing bowel obstruction is rare, and the incidence is 0.5%-5.8% [2]. The classification system devised by Ghahremani for internal herniae includes six types: paraduodenal herniae, herniae through the foramen of Winslow, transmesenteric herniae, pericaecal herniae, intersigmoid herniae, and paravesical herniae [3]. The internal hernia encountered in our case is rare and is not part of this classification.

There have only been a few reports of bowel herniating under the external iliac arteries, and all of them have occurred following lymphadenectomies for pelvic organ malignancies [1,4-9]. The patients in these reports have presented with features of bowel obstruction between two months and seven years following their lymphadenectomies and invariably have had the operations performed by minimally invasive surgical techniques - laparoscopically or using a robot.

Diagnosis of this condition on cross-sectional imaging may not be apparent since there is usually very low suspicion for such pathology. The scan of our patient revealed features of SBO with no clear point of transition. Some authors have diagnosed this condition by identifying a closed loop of the bowel at the level of the artery [4,5,7,8].

There is possibly no role for conservative management for this condition. Considering the pathology, it is unlikely that a wait and watch policy would be successful and may end up causing more harm than good since the herniated bowel might progress from a stage of incarceration to strangulation and gangrene. Barring Dumont et al. [4], who noted no evidence of bowel necrosis on laparoscopy, all other authors have performed a laparotomy in this situation, and all but two patients [4,8] have required resection of dead bowel and anastomosis. In our case, we also wanted to ensure that the ileal conduit was not a part of this pathology or damaged during the procedure. Although we noted that the conduit was not involved, a cautious approach was taken after discussing with the Urologist, and a catheter was placed in it for future imaging studies, if necessary. Another complication of this condition mentioned by Viktorin-Baier et al. [7] has been thrombosis of the external iliac artery requiring a thrombectomy and resection-anastomosis of 2 cm of the artery to reduce its redundancy.

Managing the hernial orifice is challenging, and multiple options are described. Guba Jr. et al. [10] sutured a free peritoneal graft across the aortic bifurcation and the iliac vessels to the pre-vertebral and pre-sacral fascia. In less than 24 hours, this was complicated by a pre-gangrenous change of the right lower limb, necessitating a left to right femoro-femoral by-pass using a Dacron graft. Frenzel et al. [5] managed the hernial orifice by closing it using continuous 5-0 PDS sutures. Other options discussed in the literature are fixation of the artery to the lateral abdominal wall using a fibrin sealant patch [7], using an omentum to repair the defect, placing a mesh [4] or re-positioning the artery in the retroperitoneum by closing the parietal peritoneum over it [1,8]. In our case, after discussing with the vascular surgeon, a decision was taken not to perform further procedures on the artery, especially considering contamination of the operative field with bowel content and placing sutures on the parietal peritoneum would not have been successful as it was acutely inflamed. This conservative approach has been adopted by other surgeons as well [3,9].

One preventive measure surgeons can take to avoid this condition is closing the peritoneum during the index surgery. However, this is not routinely done since these procedures are done using minimally invasive techniques. It has to be borne in mind that closing the parietal peritoneum will likely increase the incidence of symptomatic lymphocoeles and wound complications, at least theoretically, since they will not drain into the peritoneal cavity anymore.

## Conclusions

Bowel herniation under the iliac artery is rare. It may be challenging to identify this pathology on a CECT scan, but there should be a high index of suspicion, especially in a patient who has undergone pelvic lymphadenectomy. Surgical intervention will be required early, and there may be a role for a laparoscopic approach in an early stage. There is a high likelihood that these patients will require resection and anastomosis of the bowel. There is no clear consensus regarding managing the hernial orifice, and it might be a reasonable option to do nothing in case approximating the peritoneum to reposition the artery in the retroperitoneal space is not possible.
